# A novel protein encoded by circFNDC3B inhibits tumor progression and EMT through regulating Snail in colon cancer

**DOI:** 10.1186/s12943-020-01179-5

**Published:** 2020-04-02

**Authors:** Zihao Pan, Jianye Cai, Jiatong Lin, Huinian Zhou, Jingwen Peng, Jinliang Liang, Long Xia, Qi Yin, Baojia Zou, Jun Zheng, Liang Qiao, Lei Zhang

**Affiliations:** 1 Guangdong Provincial Key Laboratory of Malignant Tumor Epigenetics and Gene Regulation, Sun Yat-Sen Memorial Hospital, Sun Yat-Sen University, Guangzhou, China; 2 Department of Hepatic Surgery and Liver Transplantation Center, Department of Hepatic Surgery and Liver Transplantation Center, The Third Affiliated Hospital, Sun Yat-sen University, 600 Tianhe Road, Guangzhou, 510630 China; 3 Guangdong Key Laboratory of Liver Disease Research, Key Laboratory of Liver Disease Biotherapy and Translational Medicine of Guangdong Higher Education Institutes, The Third Affiliated Hospital, Sun Yat-sen University, 600 Tianhe Road, Guangzhou, 510630 China; 4 Department of General Surgery, Lanzhou University Second Hospital, Lanzhou, China; 5 CookGen Biosciences Center, Guangzhou, China; 6 Department of Hepatobiliary Surgery, The Fifth Affiliated Hospital, Sun Yat-sen University, Zhuhai, China; 7 Storr Liver Centre, Westmead Institute for Medical Research, University of Sydney at Westmead Hospital, Westmead, NSW 2145 Australia; 8 Department of Biliary-Pancreatic Surgery, The Third Affiliated Hospital, Sun Yat-sen University, 600 Tianhe Road, Guangzhou, 510630 China

**Keywords:** circRNA, circFNDC3B, FBP1, Colon cancer

## Abstract

**Background:**

Colon cancer (CC) is a common malignant cancer. Recently, circFNDC3B was found to exert biological function in multiple cancers. However, it was unclear whether the potential protein encoded by circFNDC3B is involved in carcinogenesis of CC.

**Methods:**

We used Sanger sequence and RNase R digestion assay to confirm the existence of circFNDC3B, and quantitative real-time PCR was used to evaluate the circRNA’s expression. Then fluorescence in situ hybridization (FISH) was performed to study location of circFNDC3B. The identification of protein encoded by circFNDC3B was performed using LC-MS/MS. The function of circFNDC3B-218aa on proliferation, invasion and migration were assessed by CCK8 assays, colony formation assays, transwell assays, wound-healing assays and animal experiments. RNA-sequencing and western blot were used to identify the gene regulated by circFNDC3B-218aa. Finally, glucose metabolism-related assays were performed to further investigate function of circFNDC3B-218aa.

**Results:**

CircFNDC3B was localized mostly in the cytoplasm, and was decreased in CC cell lines and tissues. The patients with low circFNDC3B expression had a shorter OS (*P* = 0.0014) than patients with high expression. Moreover, circFNDC3B inhibited the proliferation, invasion and migration of CC cells. Next, we identified that circFNDC3B could encode a novel protein circFNDC3B-218aa. Furthermore, circFNDC3B-218aa, not circFNDC3B, inhibited the proliferation, invasion and migration of CC. Additionally, the in vivo experiments implied that up-regulated circFNDC3B-218aa exhibited an inhibitory effect on CC progression. By RNA-sequencing, western blot and glucose metabolism-related assays, we found that circFNDC3B-218aa inhibited the expression of Snail, and subsequently promoted the tumor-suppressive effect of FBP1 in CC.

**Conclusions:**

The novel circFNDC3B-218aa may serve as a tumor suppressive factor and potential biomarker which may supply the potential therapeutic target for CC.

## Introduction

Colon cancer (CC) is a malignant cancer with increasingly high prevalence, and is the fourth leading cause of cancer-related death worldwide [1]. Although the clinical outcome of CC patients has met great improvement due to the development of novel therapeutic approaches, CC is still a deadly disease [2]. Hence, it is important to deeply explore the molecular mechanism of CC progression and to identify the potential prognosis biomarkers of CC.

Circular RNAs (circRNAs) are believed to be a group of endogenous non-coding RNAs (ncRNAs) [3, 4]. CircRNAs are circularized by the connection of a 5′ splice site with the 3′ splice site of an upstream exon or intron, which is also called back-splicing reaction [5]. Increasing evidences indicated that circRNAs are not useless byproducts created as splicing errors and that they may have regulatory roles intracellularly [6]. Acting as miRNA sponges is the most commonly reported function of circRNAs [7, 8]. Additionally, circRNAs could bind to related proteins and regulate their biological behaviors [9, 10]. Like most ncRNAs, circRNAs were previously assumed to be untranslatable because of the lack of obvious open reading frames (ORFs). However, several recent studies have reported that circRNAs could actually encode proteins [11, 12].

CircFNDC3B, originated from exons 5 and 6 of FNDC3B gene in chr3, has been recently found to be functional in previous studies [13–15]. Among these studies, Liu et al. reported that circFNDC3B is a tumor-suppressive factor in bladder cancer by sponging miR-1178-3p [13]. Another study indicated that circFNDC3B interacts with IGF2BP3 and promotes the migration and invasion of gastric cancer [15]. Although the miRNA sponging and protein binding effects of it have received some attention, there are few reports about the protein translation process of circFNDC3B. In order to explore its potential protein encoding ability, we have matched circFNDC3B with circRNADb (http://202.195.183.4:8000/circrnadb/circRNADb.php) and found a predicted ORF. However, it remains unclear whether the predicted protein encoded by circFNDC3B exists in CC and whether it exerts function in CC.

In this study, we identified that circFNDC3B was low expressed in CC. CircFNDC3B may encode a 218 amino acid novel protein which was termed the circFNDC3B-218aa. The in vitro *and* in vivo data indicated that circFNDC3B-218aa decreased proliferation, invasion and migration abilities of CC cells. Moreover, we found that circFNDC3B-218aa inhibited tumor progression and epithelial-mesenchymal transition (EMT) via alleviating the repressive effect of Snail on FBP1 in colon cancer.

## Materials and methods

### Ethical statement and tissue collection

Eighty-seven CC specimens and their paired normal tissues were obtained from patients at the Lanzhou University Second Hospital between 2013 and 2015. The tissues were immediately frozen and stored at − 80 °C after surgical resection. Paraffin-fixation was performed by experienced pathologists. The collection of all clinical data was performed after each surgery. This study was approved by the Ethics Committee of Lanzhou University Second Hospital and the written informed consent of each participant was obtained before surgery.

### Cell culture and RNase R treatment

Human CC cell lines (DLD1, HCT116, SW480, LoVo, Caco2 and HT29) and normal colon mucosal epithelial cell line (NCM460) were purchased from ATCC. All cells were cultured in DMEM supplemented with 10% FBS under standard culture conditions (5% CO_2_, 37 °C). RNase R (Epicentre Technologies, USA) was used to degrade linear mRNA. In brief, we extracted RNAs from CC cells and split RNA to two parts: one for RNase R digestion and another for control with digestion buffer only. The samples were incubated at 37 °C for 30 min. Then the expression levels of circFNDC3B and FNDC3B were detected by quantitative real-time RT-PCR.

### Total RNA extraction and quantitative real-time PCR

The extraction of total RNA sample was completed with RNAiso Plus (TaKaRa, Japan) according to manufacturer’s instructions. The concentration and purity of all samples were subsequently measured via NanoDrop 2000 (Thermo Scientific, Wilmington, DE, USA). Corresponding cDNAs were generated using PrimeScript RT Master Mix (TaKaRa, Japan). Quantitative real-time PCRs (qRT-PCR) were performed on a LightCycler® 96 System (Roche, Switzerland). The relative expression was calculated by the ΔCq method. All primer sequences were given in Additional files 1 (Table S1).

### RNA fluorescence in situ hybridization

RNA fluorescence in situ hybridization (RNA-FISH) was performed to study the location of circFNDC3B by a Fluorescent in Situ Hybridization Kit (RiboBio, Guangzhou, China) in accordance with the manufacturer’s instructions.

### Oligonucleotide transfection

circFNDC3B overexpressing vector and the control plasmid (pCDH-CMV-MCS-EF1-copGFP-Puro) were purchased from General Biosystems (General Biosystems, Anhui, China). The expression of circFNDC3B was knockdown by siRNAs generated by GenePharma (GenePharma Corporation, Shanghai, China). To verify the exist of circFNDC3B-218aa, we constructed four flag labeled vectors for circFNDC3B using lentivirus vectors (pCDH-CMV-MCS-EF1-copGFP-Puro) which were purchased from General Biosystems (General Biosystems, Anhui, China) and transfected them stably into cell lines. The full designs of sequences synthesized and cloned into vector were listed in Additional file 2. Cells were seeded in approximately 60% confluence before transfection. All siRNAs and their vectors were transfected using a lipofectamine 3000 transfection kit (Invitrogen, USA) following the manufacturer’s instructions. The transfection efficiency was assessed via qRT-PCR analysis.

### Cell proliferation assay

Cell Counting Kit-8 (CCK-8, ImmunoWay Biotechnology Company Plano, TX, USA) were performed to monitor the growth of cells. In brief, cells transfected with si-circFNDC3B or overexpression vectors were seeded into 96-well plates and cultured for 24, 48, 72 and 96 h, respectively. OD450 values were determined.

### Colony formation assay

The cloning capability of CC cells was evaluated by colony formation assays. Cells were seeded into 6-well plates and incubated for 2 weeks. After staining, the visible colonies were stained and counted.

### Transwell invasion assay

Transwell assays were used to assess the invasion ability of cells using the transwell chambers (Costar, USA), which were precoated with Matrigel. CC cells in serum-free medium were added to the upper chambers (pore size, 8 μm; Corning Inc., Tewksbury, MA, USA) and DMEM with 10% FBS was added to the lower chambers. The cells migrated into the lower chambers were fixed in 4% paraformaldehyde after incubation for 24 h and stained with crystal violet. Random fields were imaged and number of cells was counted.

### Wound-healing assay

Wound-healing assays were used to assess the migration ability of cells. The CC cells were cultured in the six-well plates until the cell confluence reached 95%. After we performed vertical scratched in the six-well plates, serum-free medium was added in it. Afterwards, image collection and migration distance measurement were conducted at 0 h and 24 h.

### Western blot analysis

Extraction of proteins was performed with RIPA buffer (CWBIO, China). The samples were electrophoresed by SDS-PAGE and transferred to nitrocellulose membranes, which were then incubated with primary antibodies at 4 °C overnight. After that, the membranes were incubated with secondary antibodies at room temperature for 1 h. Signals detection and images acquisition were performed by Immobilon ECL substrate (Millipore, Germany) and Optimax X-ray Film Processor (Protec, Germany). The antibodies used in this study were listed in Additional file 3.

### Analysis of peptide patterns by LC-MS/MS

The identification of protein was performed using LC-MS/MS. In brief, proteins were subjected to 12% SDS-PAGE gel. The protein bands near 25 kDa were excised and chopped into 1mm^3^ pieces. Peptide mixtures were extracted from the chopped gels for LC-MS/MS analysis finally.

### Metabolism assay

Intracellular ATP production was measured using a CellTiter-Glo Luminescent Cell Viability Assay kit (Promega, Madison, WI, USA). Lactate, pyruvate production and intracellular glucose uptake were measured using lactate, pyruvate and glucose assay kits (BioVision, Milpitas, CA, USA) according to the manufacturers’ instructions, respectively.

### Animal experiment

Ethical approval was approved by the Ethics Committee of Sun Yat-sen University (Guangzhou, China). All animal care and procedures were performed according to the institutional guidelines. Lentiviral vector (pCDH-CMV-MCS-EF1-copGFP-Puro) mixed with packing plasmid (psPAX2) and envelope plasmid (pMD2G) were transfected into 293 T cells using Lipofectamine 3000. Viral supernatants were collected after 48 h. Lentivirus was infected into HCT116 cells according to the manufacturer’s instructions. The stable lines (transfected respectively with empty vector, circFNDC3B, circFNDC3B-mut and circFNDC3B-218aa) were selected with Puromycin (Life Technologies). The stable transfected HCT116 cells (5 × 10^6^/200 μL PBS) were injected subcutaneously into the right backs of 4-week-old BALB/c nude mice. The size of tumors was measured every 3 days and their volumes were calculated. Thirty days later, the mice were sacrificed, and the tumors were excised for further analysis. To assess the effect of circFNDC3B-218aa on liver metastases, HCT116 cells transfected with four vectors were injected into the spleens of recipient mice, and bioluminescent images were captured. The liver metastatic nodules were excised for immunohistochemistry (IHC).

### Immunohistochemistry staining

In brief, 4-μm thick paraffin-embedded sections were prepared and received dewaxing, dehydrating and treating with 3% H_2_O_2_ at 37 °C for 10 min. After rinsed twice with PBS, the samples were boiled in ethylene diamine tetraacetic acid (EDTA, pH 8.0) at 95 °C for 20 min and cooled down to 25 °C for antigen repairment. Subsequently, the sections were treated with normal goat serum at room temperature for 10 min to block non-specific antigens and incubated with primary antibodies at 4 °C overnight, followed by incubation with the secondary antibody at 37 °C for 30 min. Finally, the sections were observed under a light microscope (Leica, Germany) after treated with diaminobenzidine. Five different fields (magnification × 400) in each section were selected to evaluate the count of positive cells.

### Statistical analysis

The data are showed as mean ± standard deviation (SD) or as values directly. As appropriate, unpaired two-tailed Student’s t-test was used to evaluate the significance of difference. Statistical analyses were conducted using Stata (version 13.1, StataCorp,College Station, TX) and GraphPad Prism 7 software (GraphPad Software, San Diego, CA). *P* value < 0.05 was considered to be statistically significant.

## Results

### The characterization of circFNDC3B in CC

**Fig. 1:**
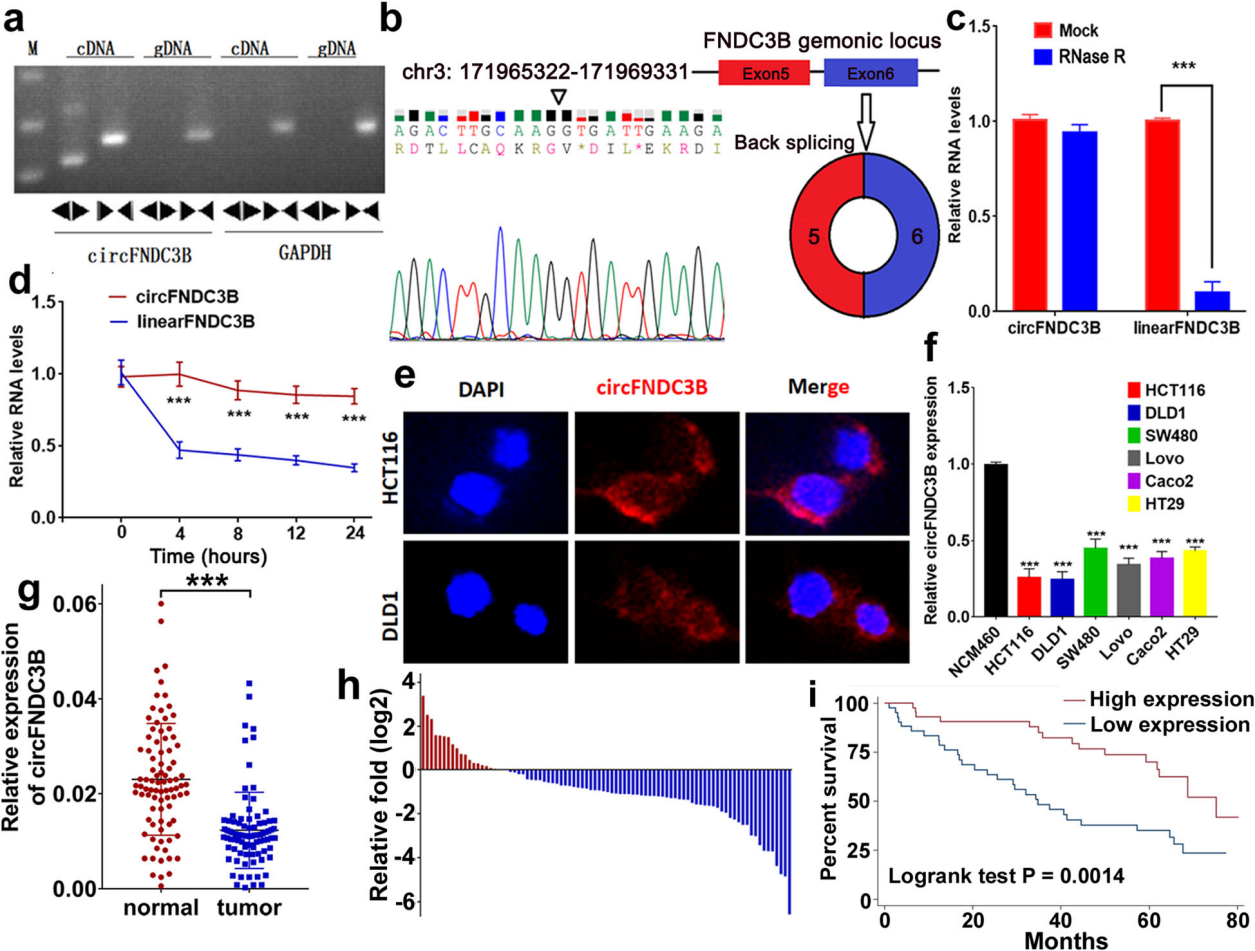
The identification and characteristics of circFNDC3B in CC. **a** Divergent primer detected circular RNAs in cDNA but not gDNA. **b** Two exons form circFNDC3B by back splicing from chromosomal region and Sanger sequencing of circFNDC3B showed the back-splice junction (∇). **c** Relative RNA level of circFNDC3B and FNCDC3B mRNA treated with RNase R. **d** Relative RNA level of circFNDC3B and FNCDC3B mRNA in different time points. **e** Fluorescence in situ hybridization assay was conducted to determine the subcellular localization of circFNDC3B. **f** The circFNDC3B expression was significantly low in CC cells. **g** The circFNDC3B expression was significantly low in CC tissues compared to normal tissues. **h** The circFNDC3B expression was significantly low in 77% CC patients. **i** Kaplan–Meier’s analyses of correlations between the circFNDC3B expression levels and OS (overall survival) of 87 CC patients. ***P < 0.001

Firstly, we designed two sets of primers. Convergent primers were used to amplify only the linear form of FNDC3B, the mRNA, while divergent primers were to amplify only the circular form, the circFNDC3B. Using cDNA and genomic DNA (gDNA) as templates, the PCR products were validated by electrophoresis. The results revealed that the single and distinct product around the expected size was amplified in the divergent primers for cDNA, but no product for gDNA (Fig. 1a). Furthermore head-to-tail splicing of the amplified circFNDC3B was confirmed by the Sanger sequencing. These results suggested that circFNDC3B was derived from exon 5 and exon 6 of the FNDC3B gene, which was consistent with the circFNDC3B data from circBase (Fig. 1b). Compared with the linear form, circFNDC3B was resistant to RNase R digestion (Fig. 1c), with a longer half-life (Fig. 1d). Further fluorescence in situ hybridization (FISH) showed that circFNDC3B was localized mostly in the cytoplasm of CC cells (Fig. 1e).

### CircFNDC3B is downregulated in CC and related to poor survival

Next, we examined the circFNDC3B expression in different CC cell lines (DLD1, HCT116, SW480, LoVo, Caco2 and HT29) using qRT-PCR analysis. CircFNDC3B was significantly lower in CC cell lines in comparison to a normal human colon mucosal epithelial cell line, the NCM460 (Fig. 1f). In addition, we examined the circFNDC3B expression in 87 pairs of CC tissues and paired normal tissues and found that circFNDC3B expression was significantly decreased in cancer tissues compared with their normal counterparts (Fig. 1g). Additionally, circFNDC3B expression was low in 77% of CC patients (67/87) (Fig. 1h). The relationship between circFNDC3B and clinicopathological features was then analyzed. The CC patients were classified into high- and low-level groups according to circFNDC3B expression (Table 1). Briefly, low circFNDC3B was positively correlated with lymphatic metastasis (*P* = 0.024). No significant correlation was observed concerning other clinicopathological features, including age, T stage, gender, and differentiation. Kaplan–Meier survival curves indicated that patients with lower circFNDC3B expression had a significantly shorter OS than patients with higher expression (*P* = 0.0014) (Fig. 1i). Furthermore, multivariate COX analysis revealed that the expression of circFNDC3B was an independent prognostic factor for CC prognosis (low expression vs high expression, HR = 2.27; 95% CI 1.19–4.32; *P* = 0.013) (Table 2).

**Table 1:** Relationship between circFNDC3B and clinical characteristics in CC patients(*N* = 87)

	Low expression		High expression		*P*
**Gender**	*N*	%	*N*	%	0.47
Male	35	81.40	33	75.00	
Female	8	18.60	11	25.00	
**T stage**					0.679
T1 + T2	11	25.58	13	29.55	
T3 + T4	32	74.42	31	70.45	
**N stage**					0.024*
N0	19	44.19	30	68.18	
N1 + N2	24	55.81	14	31.82	
**Difference**					0.174
Well	8	18.60	16	36.36	
Moderate	26	60.47	20	45.45	
Poor	9	20.93	8	18.18	
**Age** (mean ± SD)	59.74 ± 6.92		60.23 ± 7.12		0.749

*P < 0.05

**Table 2:** Univariate and multivariate Cox-regression analysis of prognostic factors for CC patients (*n* = 87)

	Univariate analysis			Multivariate analysis		
	HR	95% CI	*P*	HR	95% CI	*P*
T stage						
T1 + T2	1			1		
T3 + T4	2.28	(1.06,4.91)	0.036^*^	2.03	(0.93,4.44)	0.074
N stage						
N0	1			1		
N1 + N2	2.99	(1.63,5.53)	< 0.001^***^	2.29	(1.21,4.33)	0.011^*^
Gender						
Male	1					
Female	0.76	(0.36,1.58)	0.462	–	–	–
Differentiation						
Well	1					
Moderate	1.30	(0.66,2.56)	0.455	–	–	–
Poor	0.95	(0.38,2.40)	0.914	–	–	–
Expression of circFNDC3B						
High	1			1		
Low	2.67	(1.43,4.99)	0.002^**^	2.27	(1.19,4.32)	0.013^*^

*P < 0.05

**P < 0.01

***P < 0.001

### CircFNDC3B inhibits the proliferation, migration and invasion of CC cells

**Fig. 2:**
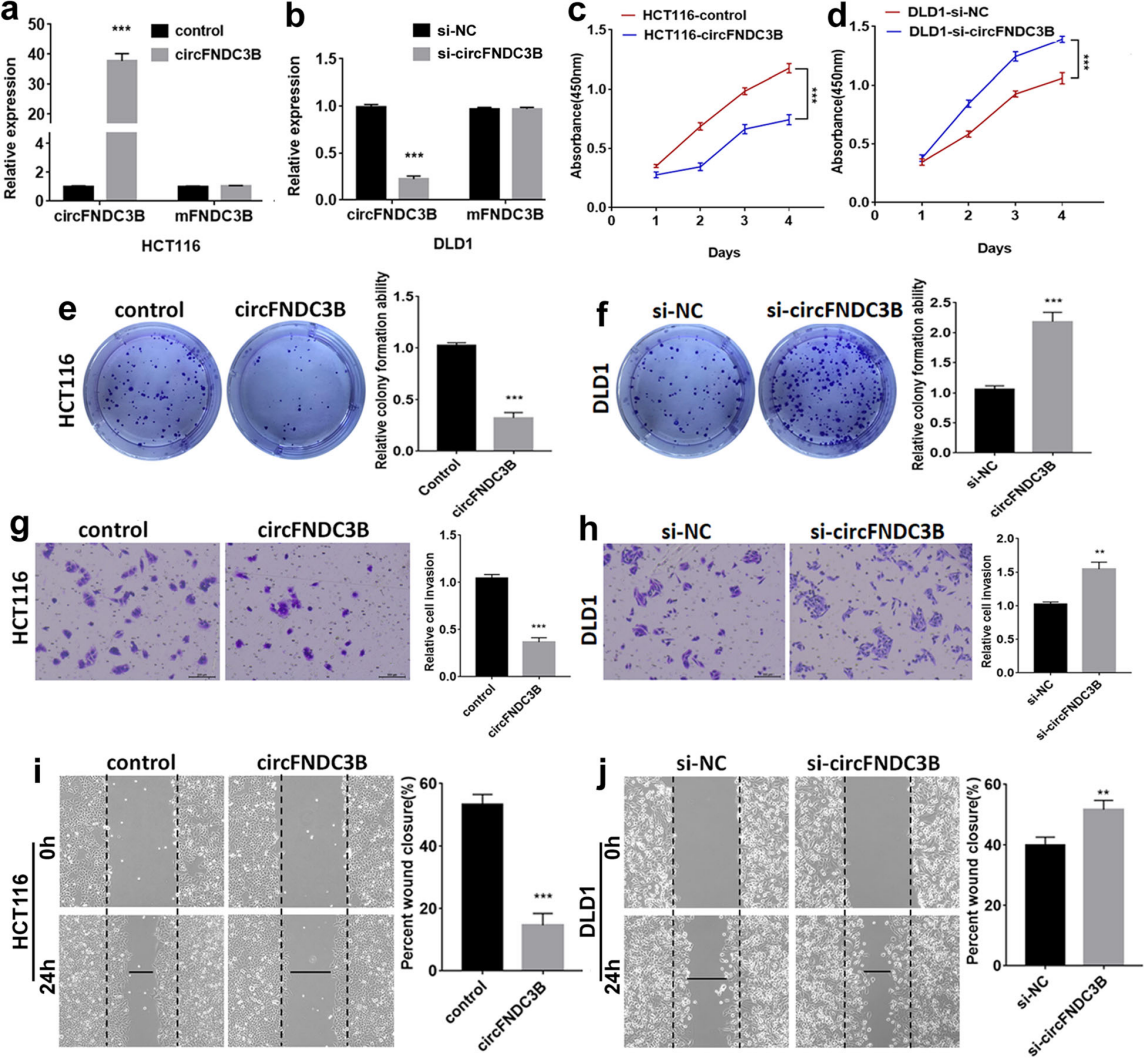
The function of circFNDC3B in CC cells. **a** Expression of circFNDC3B and FNDC3B mRNA in HCT116 cells transfected with overexpressing circFNDC3B, and **b** DLD1 cells with siRNA. **c** and **d** The effect of circFNDC3B on cell proliferation in vitro using CCK8 assay after upregulating or downregulating circFNDC3B in CC cells. **e** and **f** The effect of circFNDC3B on cell proliferation in vitro using colony formation assay after upregulating or downregulating circFNDC3B in CC cells. **g** and **h** Cell invasion abilities were assessed by transwell assay after upregulating or downregulating circFNDC3B in CC cells. **i** and **j** Cell migration abilities were assessed by wound-healing assay after upregulating or downregulating circFNDC3B in CC cells. **P < 0.01, ***P < 0.001

To investigate the biological function of circFNDC3B in CC, gain-of-function and loss-of-function assays were performed. After transfection of the circFNDC3B overexpression plasmid, there was no significant change of FNDC3B mRNA in CC cells (Fig. 2a). Otherwise, the circFNDC3B was knocked down after the cells being transfected with siRNA targeting the back-splice region, and there was no significant change in its linear counterpart, FNDC3B mRNA (Fig. 2b). CircFNDC3B upregulation significantly inhibited the proliferation of CC cells (Fig. 2c), as confirmed in the CCK8 assays. Colony formation assays also indicated that the clonality of CC cells was suppressed by up-regulating circFNDC3B (Fig. 2e). The result of transwell assays showed that invasion of CC cells was suppressed, again by circFNDC3B upregulation (Fig. 2g). Wound-healing assay showed that circFNDC3B overexpression also inhibited the migration abilities of CC cells (Fig. 2i). In contrast, downregulation of circFNDC3B yielded the reverse effects, which promoted the proliferation, invasion and migration abilities in these CC cells (Fig. 2d, f, h, and j).

### A novel protein encoded by circFNDC3B

**Fig. 3:**
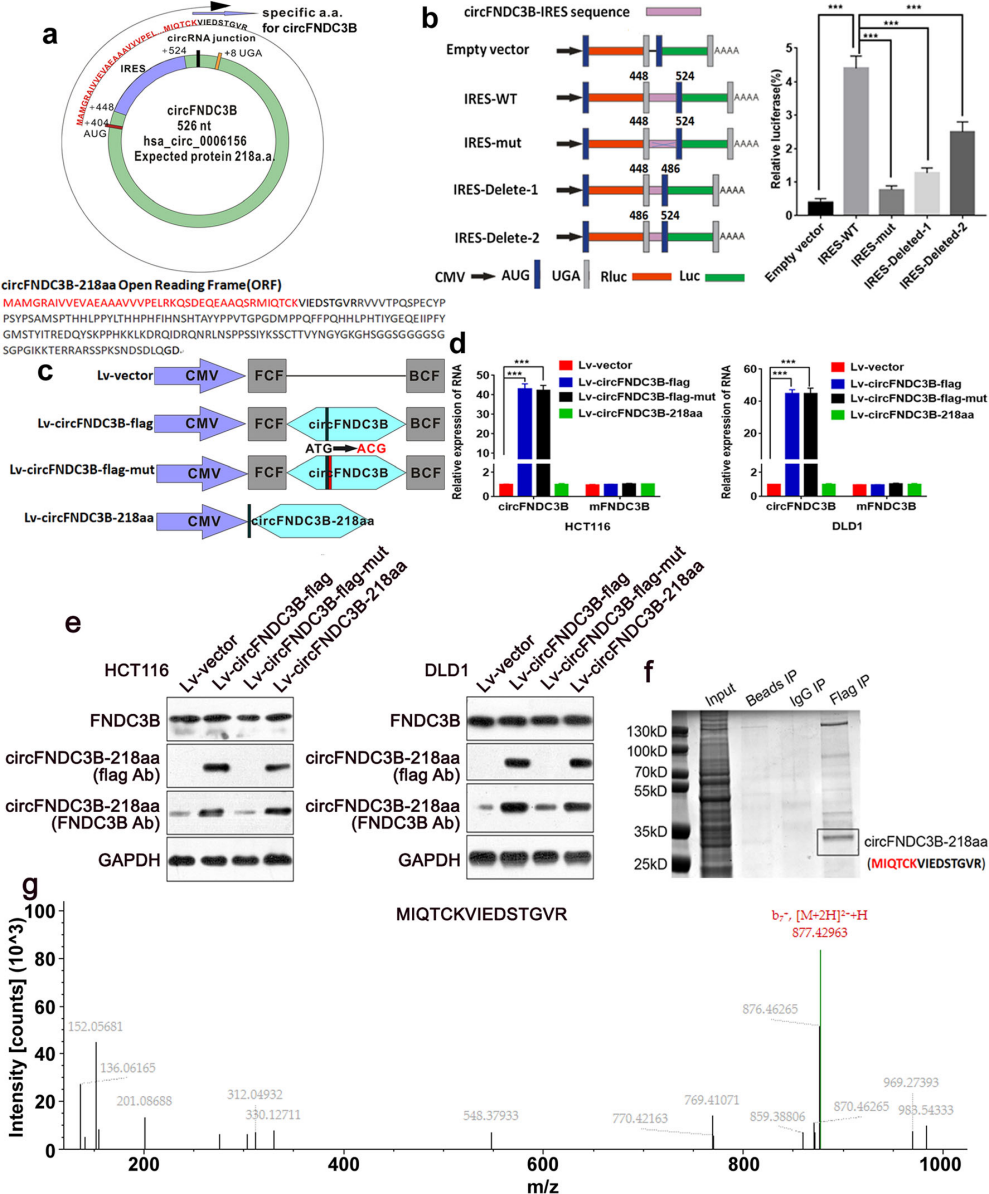
Evaluation of the coding ability of circFNDC3B. **a** Upper panel, the putative open reading frame (ORF) in circFNDC3B. The ORF implied that it took more than one whole circle of circFNDC3B. Lower panel, the sequences of putative ORF are shown. **b** The putative IRES activity of circFNDC3B was tested. Left panel, IRES sequences in circFNDC3B or its different truncation/mutation were cloned between the Rluc and Luc reporter genes with independent start (AUG) and stop (UGA) codons. Right panel, the relative luciferase activity of Luc/Rluc in the above vectors was tested. **c** Four vectors were constructed. Lv-vector; Lv-circFNDC3B-flag: flag-labeled circFNDC3B sequence was cloned into a CMV-induced expression vector; Lv-circFNDC3B-flag-mut: flag-labeled circFNDC3B sequence with start codon mutant (ATG → ACG) was cloned into a CMV-induced expression vector; Lv-circFNDC3B-218aa: flag-labeled circFNDC3B-218aa sequence was cloned into a CMV-induced expression vector. FCF and BCF are sequences which could circularize the sequence of circRNA. **d** Relative RNA expression of circFNDC3B and linearFNDC3B were detected by qRT-PCR. **e** The expression level of flag-label circFNDC3B-218aa and FNDC3B were detected by Western blotting analysis. **f** The lysates from indicated cells were separated by SDS-PAGE. Protein bands near 25 kDa were excised manually and summited for identification by LC-MS/MS. **g** circFNDC3B-218aa junction-specific peptide (MIQTCKVIEDSTGVR) was identified. ***P < 0.001

Upon the annotation of circFNDC3B from circRNA Db, an ORF named the 657-nt ORF with the potential to encode a 218-aa peptide was found. In human circFNDC3B, the tandem “AUG” within the RNA circle could start the translation of a novel protein. The ORF suggested that it took more than one whole circle of circFNDC3B to translate the putative 218 amino-acid (aa) protein. The protein contained a unique sequence and we termed it the ‘circFNDC3B-218aa’ (Fig.3a). An internal ribosomal entrance site (IRES) is required for the 5′-cap independent translation, and we found there was an IRES in the ORF (from + 448 to + 524). Furthermore, we verified the activity of the IRES in circFNDC3B by using a dual-luciferase assay, which was showed in Fig. 3b.

To verify the exist of circFNDC3B-218aa, four kinds of flag-labeled vectors were constructed (Lv-vector; Lv-circFNDC3B-flag: flag-labeled circFNDC3B sequence was cloned into a CMV-induced expression vector; Lv-circFNDC3B-flag-mut: flag-labeled circFNDC3B sequence with start codon mutant (ATG → ACG) was cloned into a CMV-induced expression vector. Lv-circFNDC3B-218aa: the flag-labeled circFNDC3B-218aa sequence was cloned into a CMV-induced expression vector) (Fig. 3c). After transfection with Lv-circFNDC3B-flag vector and Lv-circFNDC3B-flag-mut vector, the circFNDC3B expression was significantly up-regulated. In the CC cells transfected with Lv-vector and Lv-circFNDC3B-218aa, there was no obvious change in the level of circFNDC3B (Fig. 3d). All four vectors transfected did not affect the linear FNDC3B mRNA. Furthermore, the flag-labeled circFNDC3B-218aa protein was detected at approximate 25 kDa after Lv-circFNDC3B-flag or Lv-circFNDC3B-218aa transfection. Besides, the FNDC3B antibodies targeting the middle part of FNDC3B could detect circFNDC3B-218aa in Lv-vectors and Lv-circFNDC3B-flag-mut-transfected cells, while Lv-circFNDC3B-flag or Lv-circFNDC3B-218aa transfected cells showed more obvious expression of circFNDC3B-218aa at 25 kDa. Moreover, we found that these four vectors did not affect the 133Kda FNDC3B expression (Fig. 3e). After separation of the pulldown cell lysates from the cell transfected with Lv-circFNDC3B-flag, protein bands at the size of 25 kDa were excised and submitted for the LC-MS/MS (Fig. 3f). As a result, the circFNDC3B-218aa translated from circFNDC3B was confirmed, and the unique sequences of circFND3CB-218aa (MIQTCKVIEDSTGVRR) were subsequently identified (Fig. 3g). To further prove the circFNDC3B-218aa is endogenous, we used the FNDC3B antibodies which can also recognize the circFNDC3B-218aa with their same amino acids sequence to conduct co-immunoprecipitation in the extracts from the cells without forced expression. The LC-MS/MS successfully identified the specific peptide fragments from circFNDC3B-218aa (MAMGRAIVVEVAEAAAVVVPELRK) (Additional file 4 Fig.S1 a and b). Taken together, circFNDC3B could encode a novel protein in CC.

### CircFNDC3B-218aa, not circFNDC3B inhibits the proliferation, invasion and migration abilities of CC in vitro and in vivo

**Fig. 4:**
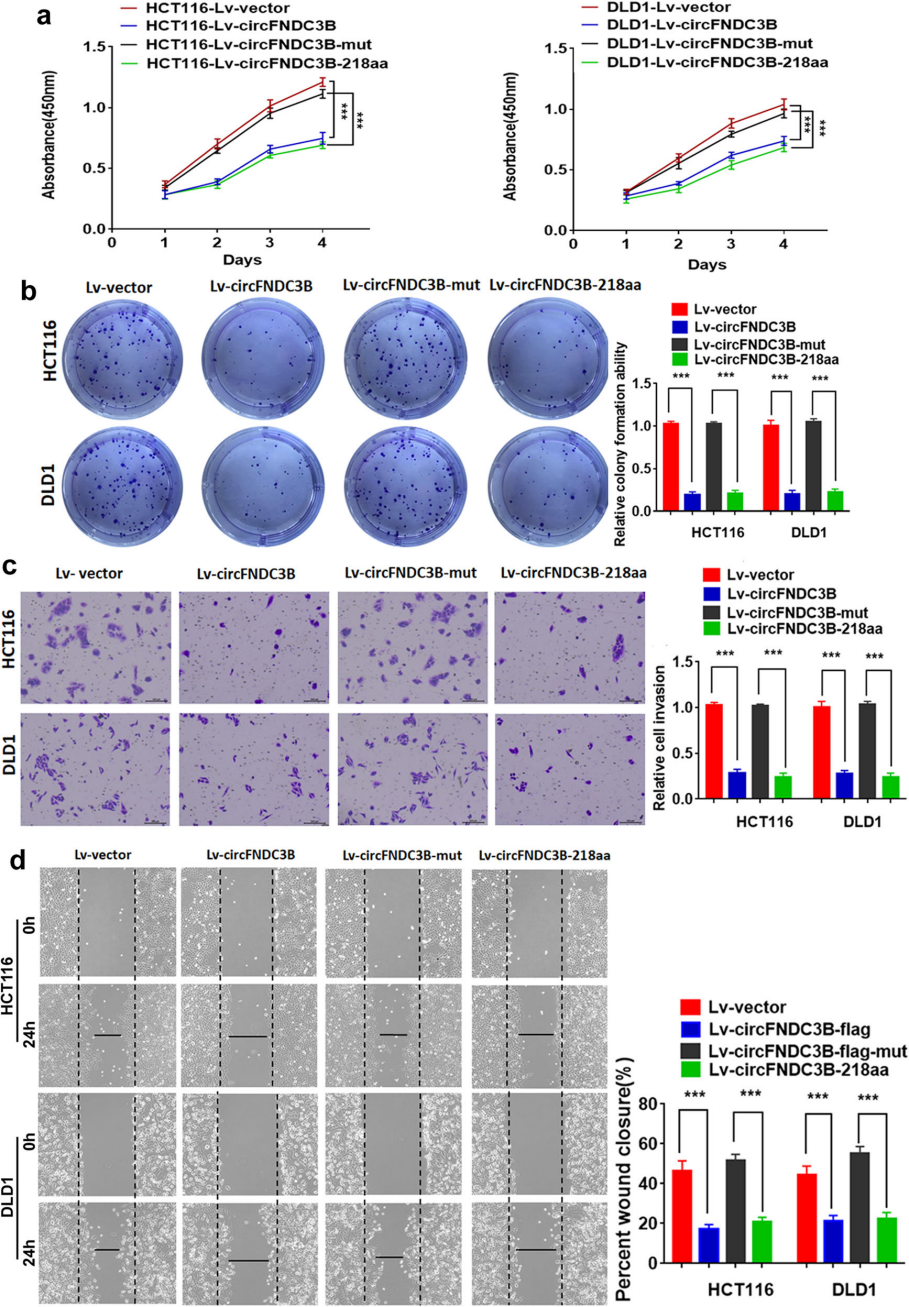
CircFNDC3B-218aa, not circFNDC3B, inhibits the proliferation, invasion and migration of CC. **a** CCK8 assays of HCT116 and DLD1 transfected with four vectors. **b** Colony formation assays of HCT116 and DLD1 transfected with four vectors. **c** Transwell invasion assays of HCT116 and DLD1 transfected with four vectors. **d** Wound-healing assays of HCT116 and DLD1 transfected with four vectors. ***P < 0.001

**Fig. 5:**
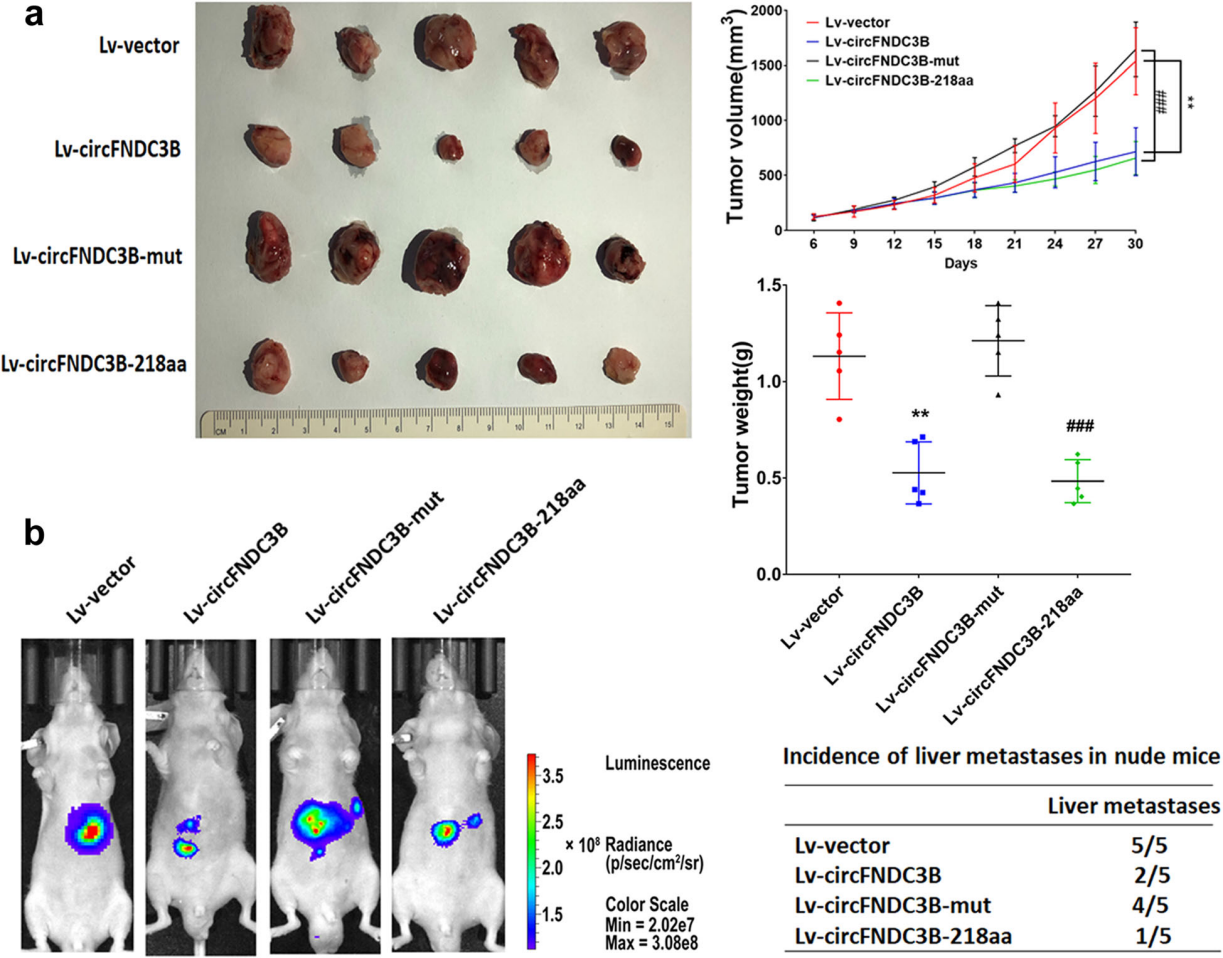
CircFNDC3B-218aa, not circFNDC3B inhibits the in vivo tumorigenicity ability of CC using animal experiments. **a** Nude mice xenografts were formed by HCT116 cells transfected with Lv-vector, Lv-circFNDC3B, Lv-circFNDC3B-mut and Lv-circFNDC3B-218aa, respectively. Tumor volumes were monitored with a caliper during the time course of 30 days, and tumor weights were also measured at the end of this study. **b** Representative BLI of the four groups is shown. The incidences of liver metastases in each group were shown. **P < 0.01 vs Lv-vector group; ### *P* < 0.001 vs Lv-circFNDC3B-mut group

To explore the biological function of circFNDC3B-218aa, the four vectors were transfected into CC cell lines. The results of CCK8 and colony formation assays indicated that the proliferation abilities of CC cells were inhibited by transfection with Lv-circFNDC3B or Lv-circFNDC3B-218aa. However, there was no effect on the proliferation in the CC cells transfected with Lv-circFNDC3B-mut which could not be translated into the circFNDC3B-218aa. The results suggested that only overexpression of circFNDC3B-218aa decreased the proliferation abilities (Fig. 4a and b). In transwell assays and wound-healing assays, overexpression of circFNDC3B-218aa inhibited the invasion and migration abilities of CC cells. However, mutant circFNDC3B did not influence the invasion and migration abilities of CC cells (Fig. 4c and d). Collectively, the above results showed that the protein form (circFNDC3B-218aa), not the circle RNA form (circFNDC3B), inhibits the proliferation, invasion and migration abilities of CC. To further assess whether circFNDC3B-218aa exerts tumor-suppressing effect in vivo, a xenograft mouse model was established by subcutaneous injection of HCT116 cells (*n* = 5 for each group). The results indicated that the growth rates and tumor weight were significantly lower in the Lv-circFNDC3B and Lv-circFNDC3B-218aa group than in the vector control and the Lv-circFNDC3B-mut group (Fig. 5a). Additionally, in vivo metastatic assay showed that Lv-circFNDC3B and Lv-circFNDC3B-218aa decreased detectable liver metastasis and the incidence of liver metastasis compared with the Lv-vector group and Lv-circFNDC3B-mut group (Fig. 5b).

### Effect of CircFND3CB-218aa on CC cells and the underlying mechanism

**Fig. 6:**
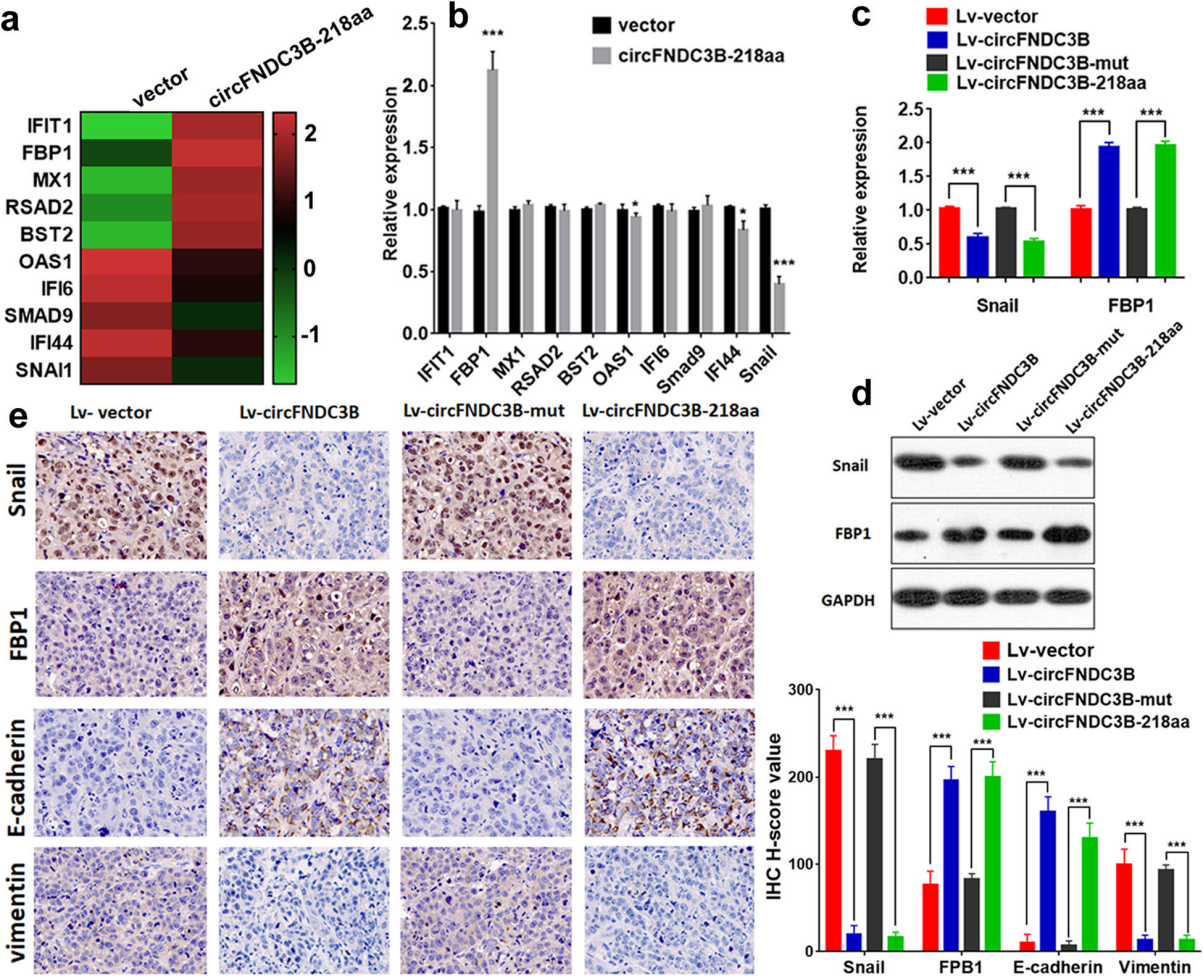
circFNDC3B-218aa regulated Snail and FBP1. **a** Heat map showed the top 10 significantly upregulated or downregulated differential genes expression between two groups. **b** qRT-PCR was used to evaluate the expression of the top 10 dysregulated genes. **c** qRT-PCR was used to evaluate the expression of Snail and FBP1 regulated by the four vectors. **d** Western blot was used to evaluate the expression of Snail and FBP1 regulated by the four vectors. **e** IHC analysis of Snail, FBP1, E-cadherin and vimentin in liver metastatic nodules. *P < 0.05, ***P < 0.001

To explore the potential regulatory mechanisms influenced by circFNDC3B-218aa in CC cell lines, we performed RNA-Seq analyses. Differentially expressed genes (DEGs) in Lv-vector and Lv-circFNDC3B-218aa groups were analyzed (Additional file 5), and Fig. 6a listed the top 10 significant DEGs. We further evaluated the gene expression through qRT-PCR, and found that four of these genes were indeed differentially expressed (Fig. 6b). Among these genes, there was few studies reported that OAS1 and IFI44 were related to tumor progression [16, 17]. Additionally, Snail was downregulated and FBP1 was upregulated in circFNDC3B-218aa group. Further qRT-PCR and western blot results showed that the Snail level was decreased and the FBP1 level was increased when the CC cells were transfected with Lv-circFNDC3B or Lv-circFNDC3B-218aa (Fig. 6c and d). According to reported articles, Snail could induce EMT via inhibiting the expression of FBP1 [18, 19]. Therefore, we speculated that circFNDC3B-218aa exerts a tumor-related role in Snail/FBP1/EMT axis. We assessed the expression of Snail, FBP1, E-cadherin and vimentin in the liver metastases specimens of nude mice. The IHC results showed that the expression levels of FBP1 and E-cadherin were significantly increased, while Snail and vimentin were decreased in the Lv-circFNDC3B and Lv-circFNDC3B-218aa group compared with the control and the Lv-circFNDC3B-mut group (Fig. 6e). Such results further revealed that circFNDC3B-218aa exhibited inhibitory effects on CC EMT and regulated the expression of Snail and FBP1.

**Fig. 7:**
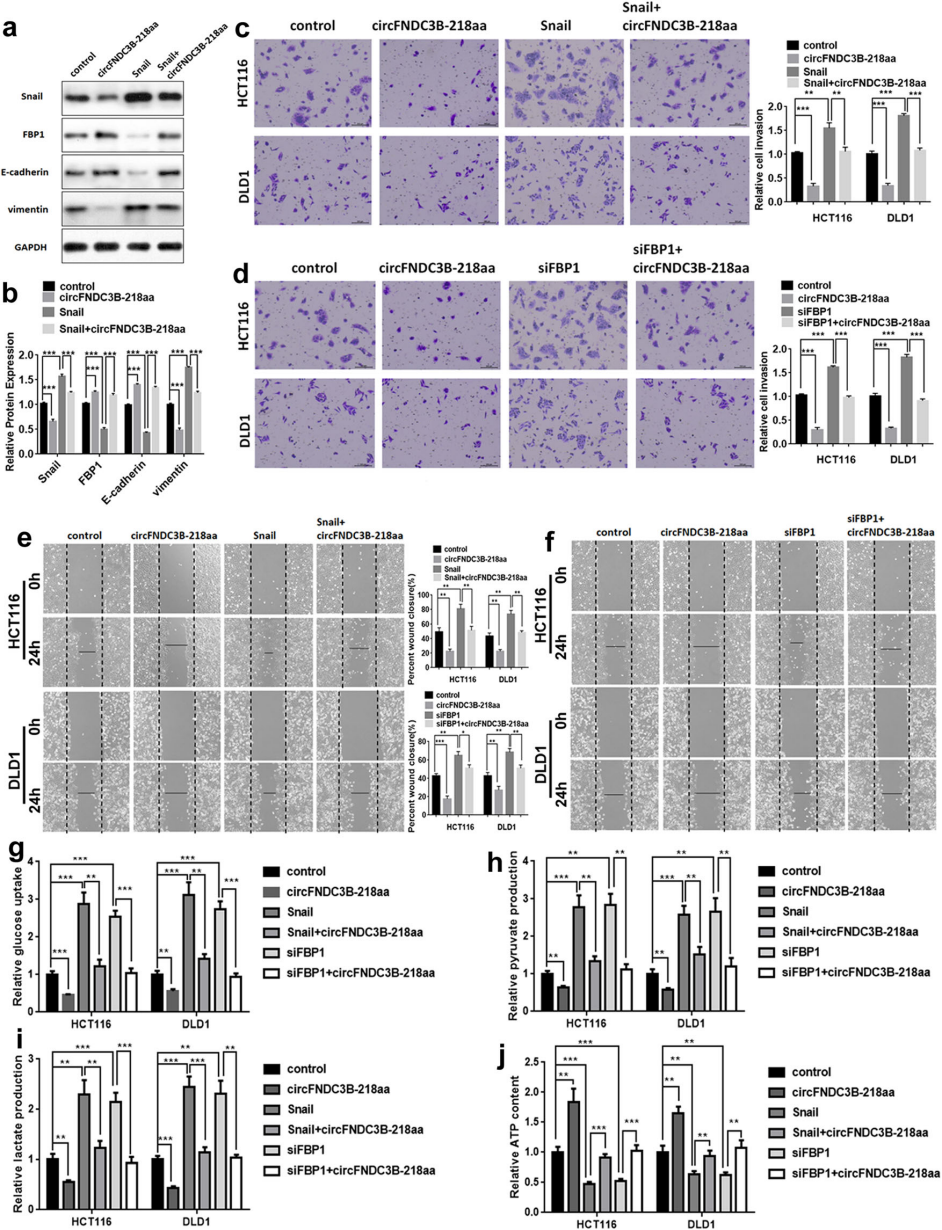
circFNDC3B-218aa promotes the tumor-suppressive effect of FBP1 in CC via inhibiting Snail. **a** The expression of levels of FBP1 and E-cadherin were inhibited by Snail, but this effect was restored by circFNDC3B-218aa presented in Western blot analysis. **b** The relative protein expression of Snail, FBP1, E-cadherin and vimentin as detected by Western blot analysis. **c** The invasion abilities enhanced by Snail were restored after co-transfection with circFNDC3B-218aa using transwell assay. **d** The invasion abilities enhanced by downregulated FBP1 were restored after co-transfection with circFNDC3B-218aa using transwell assay. **e** The migration abilities enhanced by Snail were restored after co-transfection with circFNDC3B-218aa using wound-healing assay. **f** The migration abilities enhanced by downregulated FBP1 were restored after co-transfection with circFNDC3B-218aa using wound-healing assay. **g** The glucose uptake, **h** pyruvate production, **i** lactate production, **j** ATP status in HCT116 and DLD1 cell lines. **P < 0.01, ***P < 0.001

Furthermore, we investigated the roles of circFNDC3B-218aa, FBP1 and Snail in EMT. Further Western blot analysis indicated that compared with the control group, the expression of E-cadherin and FBP1 were significantly decreased, whereas that of vimentin was increased in the HCT116 cells with overexpressed Snail. However, the above phenomenon was restored after overexpressing circFNDC3B-218aa in upregulated Snail HCT116 (Fig. 7a and b). Moreover, in transwell invasion assays and wound-healing assays, we found that the invasion and migration abilities of CC cells enhanced by Snail could be alleviated by circFNDC3B-218aa (Fig. 7c and e), suggesting the overexpression of circFNDC3B-218aa restores the oncogenic roles of Snail in CC cells. Given that FBP1 could inhibit EMT and circFNDC3B-218aa could regulate FBP1, it suggested that circFNDC3B-218aa inhibited EMT by enhancing FBP1. To investigate whether circFNDC3B-218aa could inhibit cancer progression by enhancing FBP1, we transfected CC cells with siFBP1 and circFNDC3B-218aa overexpression plasmid. Analogously, the transwell assays and wound-healing assays showed that cell invasion and migration ability were promoted by FBP1 downregulation, but were partially restored by transfection of circFNDC3B-218aa (Fig. 7d and f). These results illustrated that circFNDC3B-218aa reversed the effect of downregulated FBP1.

**Fig. 8:**
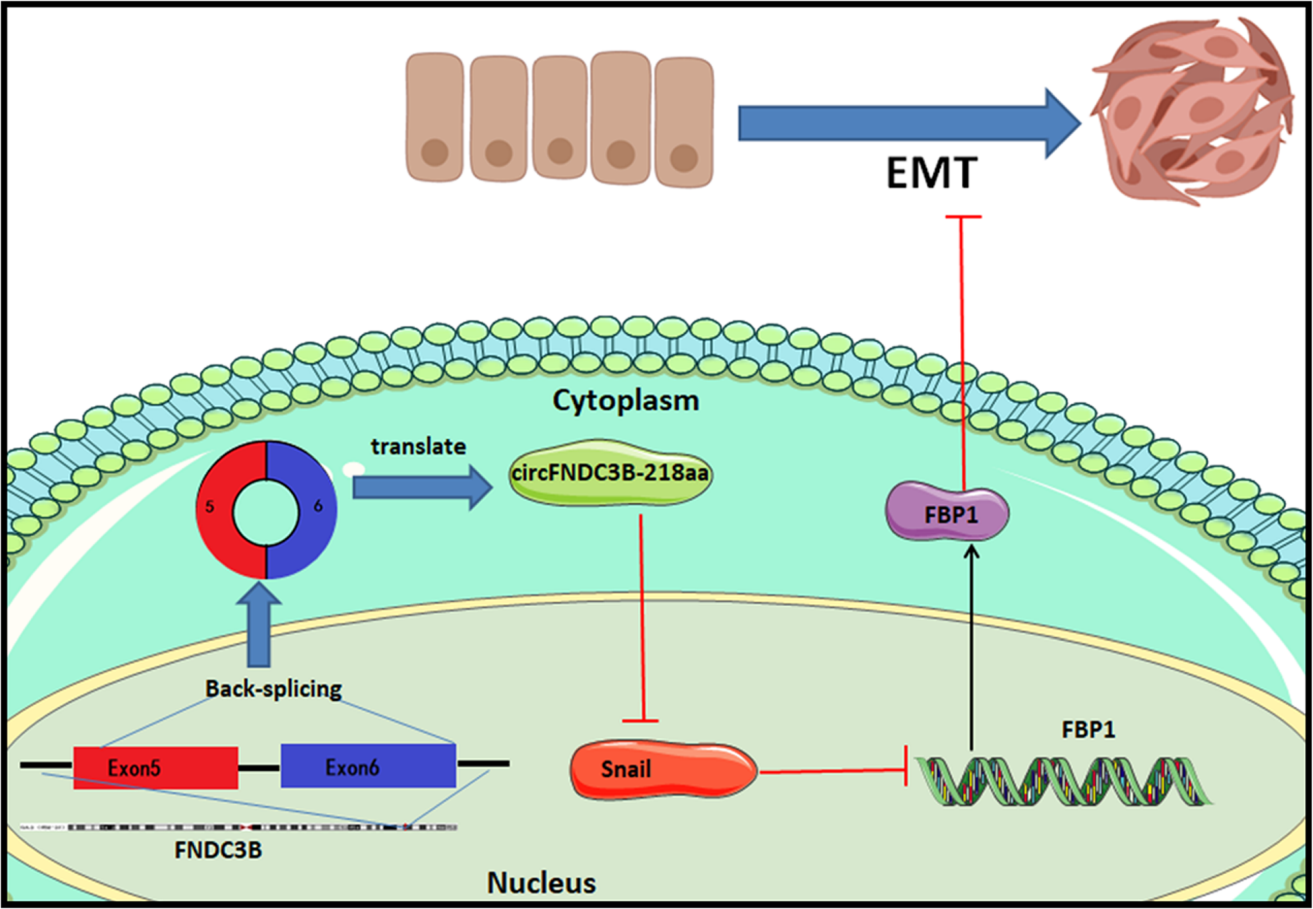
Illustration of circFNDC3B-218aa function. CircFNDC3B-218aa inhibits the expression of Snail, subsequently promotes the EMT-suppressive effect of FBP1

As a gluconeogenesis regulatory enzyme, FBP1 had been further demonstrated play an important role in impairing aggressive phenotype in various cancers via a metabolic switch from glycolysis to oxidative phosphorylation (OXPHOS) [20, 21]. Therefore, we investigated whether circFNDC3B-218aa exhibited an inhibitory effect on EMT through FBP1 participating in Warburg effect inhibition. The glucose uptake, pyruvate production and lactate production were remarkably increased in HCT116 and DLD1 cell lines with upregulated Snail or downregulated FBP1, which could be reversed by circFNDC3B-218aa overexpression (Fig. 7g-i). In addition, circFNDC3B-218aa could also elevate ATP produced by OXPHOS upon Snail upregulation or FBP1 silencing in CC cells (Fig. 7j). These results indicated that circFNDC3B-218aa enhanced metabolic reprogramming from glycolysis to oxidative phosphorylation via inhibiting Snail-FBP1 signaling axis, subsequently suppressed the EMT progression in CC cells. Taken together, these data suggested that circFNDC3B-218aa could inhibit the cancer progression and EMT via alleviating the repressive effect of Snail on FBP1 (Fig. 8).

## Discussion

CircRNAs are novel RNA molecules, with various biological functions. Among them, acting as a miRNA sponge and interaction with related proteins are the two common function patterns of circRNA. A previous study has reported that artificial circRNA was translatable in eukaryotic cells [22]. Recently, it is also reported that circRNAs translation could be driven by N6-methyladenosine [12]. Meanwhile, the circZNF609 and the circMbl have been found to be protein-encoding in two other studies [23, 24]. These reported evidences all suggested that endogenous circRNAs have the potential to encode protein. Moreover, several protein-encoding circRNAs have been identified to be involved in cancer progression. For example, a functional protein encoded by circFBXW7 could repress glioma tumorigenesis [25]. In the present study, we found that there is an ORF in the circFNDC3B sequence. Further experiments showed that circFNDC3B could encode a novel protein which suppressed colon cancer progression and EMT in vitro and in vivo.

According to previous studies, there are two main reasons why it is difficult to detect the circRNA-translated protein. One is that the protein encoded by circRNAs may overlap with the linear mRNA of the translated products. The other is that the linear mRNA products obscures the protein translated by circRNA because the circRNA level was low compared with their linear counterparts [26]. With the development of testing approaches, growing number of peptides encoded by circRNAs have been detected, and their potential biological functions have also been identified. CircRNA encoded proteins usually show the functional independence with their host gene products. For example, it has been reported that circPPP1R12A-73aa promotes CC progression via Hippo-YAP signaling [27]. In this study, we found that circFNDC3B-218aa, rather than circFNDC3B, regulated the expression of Snail and FBP1. It suggested that circFNDC3B-218aa exerted biological function in CC independently, which is similar to previous studies. In contrast, some products encoded by circRNAs are involved in their host gene functions. For example, circSHPPH-146aa prevents its host gene SHPPR from degradation [26] and circFBXW7-185aa induced c-Myc degradation [25]. Because circFNDC3B-218aa shares the majority of its amino acids sequence with that of FNDC3B with the exception of the unique amino acids in the ORF, it is possible that circFNDC3B-218aa involved in the functions of FNDC3B. However, the underlying mechanism is needed further validation.

In present study, we found that circFNDC3B-218aa could suppress the Snail expression and subsequently upregulate FBP1 and inhibit EMT in CC. Therefore, we speculated that circFNDC3B-218aa exerted its tumor-suppressive effect in Snail/FBP1/EMT axis. FBP1, one of the related-limiting enzymes in gluconeogenesis, plays an important role in glucose metabolism [19]. It had been widely reported that metabolic shifts control EMT progression and trigger tumor malignancy, especially an enhanced glycolytic phenotype switched from OXPHOS, which was called Warburg effect [28–30]. This specific tumor hallmark not only provides cancer cells with nutrients, but also constructs a more acidic tumor environment that leads to extracellular matrix destruction and induces metastasis [31, 32]. In our study, we found that circFNDC3B-218aa suppressed tumor EMT progression was mainly dependent on inhibiting of Snail expression, subsequently enhancing FBP1-induced OXPHOS. Consistently, Dong et al. demonstrated that loss of FBP1 by Snail-mediated repression was required for EMT induction via glycolytic shift [18]. Thus, disruption of glycolysis process might be a potential therapeutic target for circFNDC3B/circFNDC3B-218aa deficient colon cancer.

## Conclusions

Taken together, we found that the expression of circFNDC3B was decreased in CC tissues and cells. Additionally, the results showed that circFNDC3B could encode circFNDC3B-218aa. The circFNDC3B-218aa, not circFNDC3B itself, inhibited the proliferation and invasion of CC cells in vitro and in vivo. Furthermore, we speculated that circFNDC3B-218aa could inhibit the cancer progression and EMT via alleviating the repressive effect of Snail on FBP1. Collectively, this study expanded upon the understanding of the coding potential of circRNAs. The novel circFNDC3B-218aa is a potential biomarker which may supply the potential therapeutic target for CC.

## Supplementary information

**Additional file 1: Table S1.** The primer sequences used in this study.

**Additional file 2.** The full design of vectors was listed.

**Additional file 3: Table S2.** Antibodies used in this study

**Additional file 4: Figure S1. a** The lysates from indicated cells were separated by SDS-PAGE. Protein at 25 kDa were excised manually and summited for identification by LC-MS/MS. **b** circFNDC3B-218aa specific peptide (MAMGRAIVVE VAEAAAVVVPELRK) was identified.

**Additional file 5.** DEGs were screened.

## Data Availability

All data in our study are availability upon request.

## Abbreviations

circRNA: Circular RNA
CC: Colon cancer
OS: Overall survival
ORF: Open reading frame
EMT: Epithelial-mesenchymal transition
FISH: Fluorescence in situ hybridization
IHC: Immunohistochemistry
IRES: Internal ribosomal entrance site
FCF: Front-circular frame
BCF: Back-circular frame
