# Supplementary material for: A novel protein encoded by circFNDC3B inhibits tumor progression and EMT through regulating Snail in colon cancer
Source: Mol Cancer. 2020 Apr 2;19:71. doi: 10.1186/s12943-020-01179-5 (PMC7114813; doi:10.1186/s12943-020-01179-5)
Supplement: Supplementary file 1 — Additional file 1: Table S1. The primer sequences used in this study. [file 12943_2020_1179_MOESM1_ESM.docx]

**Table S1** The primer sequences used in this study

| Primer name | Primer sequence |
| --- | --- |
| Primers for real-time PCR |  |
| circFNDC3B | F: TTCAGACTTGCAAGGTGATTGAAG |
|  | R: ATACTGTTGTGCAGCTGCTTTT |
| liner FNDC3B | F: ACTGAAAGACCGCCAGATCG |
|  | R: TCTTGCTCGTCGCTCTGTTT |
| GAPDH | F: GGAGCGAGATCCCTCCAAAAT |
|  | R: GGCTGTTGTCATACTTCTCATGG |
| divergent GAPDH | F: GAAGGTGAAGGTCGAGTC |
|  | R: GAAGATGGTGATGGGATTTC |
| Snail | F: TGCCCTCAAGATGCACATCCGA |
|  | R: GGGACAGGAGAAGGGCTTCTC |
| FBP1 | F: GATTGCCTTGTGTCCGTTG |
|  | R: TGCCATACAGTGCGTAGCC |
| IFIT1 | F: GCGCTGGGTATGCGATCTC |
|  | R: CAGCCTGCCTTAGGGGAAG |
| MX1 | F: GTTTCCGAAGTGGACATCGCA |
|  | R: CTGCACAGGTTGTTCTCAGC |
| RSAD2 | F: TGGGTGCTTACACCTGCTG |
|  | R: GAAGTGATAGTTGACGCTGGTT |
| BST2 | F: CACACTGTGATGGCCCTAATG |
|  | R: GTCCGCGATTCTCACGCTT |
| OAS1 | F: TGTCCAAGGTGGTAAAGGGTG |
|  | R: CCGGCGATTTAACTGATCCTG |
| IFI6 | F: GGTCTGCGATCCTGAATGGG |
|  | R: TCACTATCGAGATACTTGTGGGT |
| SMAD9 | F: ATGTGATTTACTGTCGCGTGT |
|  | R: GGCGGTAGTGGTAAGGGTTAAT |
| IFI44 | F: GGTGGGCACTAATACAACTGG |
|  | R: CACACAGAATAAACGGCAGGTA |
