# Supplementary material for: A novel protein encoded by circFNDC3B inhibits tumor progression and EMT through regulating Snail in colon cancer
Source: Mol Cancer. 2020 Apr 2;19:71. doi: 10.1186/s12943-020-01179-5 (PMC7114813; doi:10.1186/s12943-020-01179-5)
Supplement: Supplementary file 2 — Additional file 2. The full design of vectors was listed. [file 12943_2020_1179_MOESM2_ESM.docx]

1.overxpressing-circ-FNDC3B

AGTGCTGAGATTACAGGCGTGAGCCACCACCCCCGGCCCACTTTTTGTAAAGGTACGTACTAATGACTTTTTTTTTATACTTCAGGTGATTGAAGATAGTACTGGAGTCCGCCGGGTGGTGGTCACACCCCAGTCTCCTGAGTGTTATCCCCCAAGCTACCCCTCAGCCATGTCTCCAACCCATCATCTCCCTCCCTATCTGACTCACCATCCACATTTTATTCATAACTCACACACGGCTTACTACCCACCTGTTACCGGACCTGGAGATATGCCGCCTCAGTTTTTTCCCCAGCATCATCTTCCCCACACAATATATGGTGAGCAAGAAATTATACCATTTTATGGAATGTCAACCTACATCACCCGAGAAGACCAGTACAGCAAGCCTCCGCACAAAAAACTGAAAGACCGCCAGATCGATCGCCAGAACCGCCTCAACAGCCCTCCTTCTTCTATCTACAAAAGCAGCTGCACAACAGTATACAATGGCTATGGGAAGGGCCATAGTGGTGGAAGTGGCGGAGGCGGCAGCGGTAGTGGTCCCGGAATTAAGAAAACAGAGCGACGAGCAAGAAGCAGCCCAAAGTCGAATGATTCAGACTTGCAAGGTAAGAAGCAAGGAAAAGAATTAGGCTCGGCACGGTAGCTCACACCTGTAATCCCAGCA

2.Lv-circ-FNDC3B-FLAG

AGTGCTGAGATTACAGGCGTGAGCCACCACCCCCGGCCCACTTTTTGTAAAGGTACGTACTAATGACTTTTTTTTTATACTTCAGGTGATTGAAGATGACTACAAAGACCATGACGGTGATTATAAAGATCATGACATCGATTACAAGGATGACGATGACAAGAGTACTGGAGTCCGCCGGGTGGTGGTCACACCCCAGTCTCCTGAGTGTTATCCCCCAAGCTACCCCTCAGCCATGTCTCCAACCCATCATCTCCCTCCCTATCTGACTCACCATCCACATTTTATTCATAACTCACACACGGCTTACTACCCACCTGTTACCGGACCTGGAGATATGCCGCCTCAGTTTTTTCCCCAGCATCATCTTCCCCACACAATATATGGTGAGCAAGAAATTATACCATTTTATGGAATGTCAACCTACATCACCCGAGAAGACCAGTACAGCAAGCCTCCGCACAAAAAACTGAAAGACCGCCAGATCGATCGCCAGAACCGCCTCAACAGCCCTCCTTCTTCTATCTACAAAAGCAGCTGCACAACAGTATACAATGGCTATGGGAAGGGCCATAGTGGTGGAAGTGGCGGAGGCGGCAGCGGTAGTGGTCCCGGAATTAAGAAAACAGAGCGACGAGCAAGAAGCAGCCCAAAGTCGAATGATTCAGACTTGCAAGGTAAGAAGCAAGGAAAAGAATTAGGCTCGGCACGGTAGCTCACACCTGTAATCCCAGCA

3.Lv-circ-FNDC3B-FLAG(MUT)

AGTGCTGAGATTACAGGCGTGAGCCACCACCCCCGGCCCACTTTTTGTAAAGGTACGTACTAATGACTTTTTTTTTATACTTCAGGTGATTGAAGATGACTACAAAGACCATGACGGTGATTATAAAGATCATGACATCGATTACAAGGATGACGATGACAAGAGTACTGGAGTCCGCCGGGTGGTGGTCACACCCCAGTCTCCTGAGTGTTATCCCCCAAGCTACCCCTCAGCCATGTCTCCAACCCATCATCTCCCTCCCTATCTGACTCACCATCCACATTTTATTCATAACTCACACACGGCTTACTACCCACCTGTTACCGGACCTGGAGATATGCCGCCTCAGTTTTTTCCCCAGCATCATCTTCCCCACACAATATATGGTGAGCAAGAAATTATACCATTTTATGGAATGTCAACCTACATCACCCGAGAAGACCAGTACAGCAAGCCTCCGCACAAAAAACTGAAAGACCGCCAGATCGATCGCCAGAACCGCCTCAACAGCCCTCCTTCTTCTATCTACAAAAGCAGCTGCACAACAGTATACAACGGCTATGGGAAGGGCCATAGTGGTGGAAGTGGCGGAGGCGGCAGCGGTAGTGGTCCCGGAATTAAGAAAACAGAGCGACGAGCAAGAAGCAGCCCAAAGTCGAATGATTCAGACTTGCAAGGTAAGAAGCAAGGAAAAGAATTAGGCTCGGCACGGTAGCTCACACCTGTAATCCCAGCA

4.Lv-circ-FNDC3B-218aa-FLAG

ATGGCTATGGGAAGGGCCATAGTGGTGGAAGTGGCGGAGGCGGCAGCGGTAGTGGTCCCGGAATTAAGAAAACAGAGCGACGAGCAAGAAGCAGCCCAAAGTCGAATGATTCAGACTTGCAAGGTGATTGAAGATAGTACTGGAGTCCGCCGGGTGGTGGTCACACCCCAGTCTCCTGAGTGTTATCCCCCAAGCTACCCCTCAGCCATGTCTCCAACCCATCATCTCCCTCCCTATCTGACTCACCATCCACATTTTATTCATAACTCACACACGGCTTACTACCCACCTGTTACCGGACCTGGAGATATGCCGCCTCAGTTTTTTCCCCAGCATCATCTTCCCCACACAATATATGGTGAGCAAGAAATTATACCATTTTATGGAATGTCAACCTACATCACCCGAGAAGACCAGTACAGCAAGCCTCCGCACAAAAAACTGAAAGACCGCCAGATCGATCGCCAGAACCGCCTCAACAGCCCTCCTTCTTCTATCTACAAAAGCAGCTGCACAACAGTATACAATGGCTATGGGAAGGGCCATAGTGGTGGAAGTGGCGGAGGCGGCAGCGGTAGTGGTCCCGGAATTAAGAAAACAGAGCGACGAGCAAGAAGCAGCCCAAAGTCGAATGATTCAGACTTGCAAGGTGATGACTACAAAGACCATGACGGTGATTATAAAGATCATGACATCGATTACAAGGATGACGATGACAAGTGA
