# Supplementary material for: A novel protein encoded by circFNDC3B inhibits tumor progression and EMT through regulating Snail in colon cancer
Source: Mol Cancer. 2020 Apr 2;19:71. doi: 10.1186/s12943-020-01179-5 (PMC7114813; doi:10.1186/s12943-020-01179-5)
Supplement: Supplementary file 3 — Additional file 3: Table S2. Antibodies used in this study [file 12943_2020_1179_MOESM3_ESM.docx]

**Table S2** Antibodies used in this study

| Antigens | Manufacturers | Applications |
| --- | --- | --- |
| FBP1 | ab109020, Abcam, Cambridge, MA, USA | 1:1000 for WB |
|  |  | 1:100 for IHC |
| E-Cadherin | #3195, Cell Signaling Technology, Beverly, MA, USA | 1:1000 for WB  1:400 for IHC |
| Vimentin | ab8978, Abcam, Cambridge, MA, USA | 1:1000 for WB  1:200 for IHC |
| Snail | ab180714, Abcam, Cambridge, MA, USA | 1:1000 for WB |
|  |  | 1:200 for IHC |
| FNDC3B | NBP1-90495,Novus Biologicals,USA | 1:1000 for WB |
|  |  |  |
| GAPDH | #5174, Cell Signaling Technology, Beverly, MA, USA | 1:2000 for WB |
| HRP-linked anti-rabbit IgG | #7074, Cell Signaling Technology, Beverly, MA, USA | 1:10000 for WB |
| HRP-linked anti-mouse IgG | #7076, Cell Signaling Technology, Beverly, MA, USA | 1:10000 for WB |
