# Supplementary figures and images for: A novel protein encoded by circFNDC3B inhibits tumor progression and EMT through regulating Snail in colon cancer
Source: Mol Cancer. 2020 Apr 2;19:71. doi: 10.1186/s12943-020-01179-5 (PMC7114813; doi:10.1186/s12943-020-01179-5)

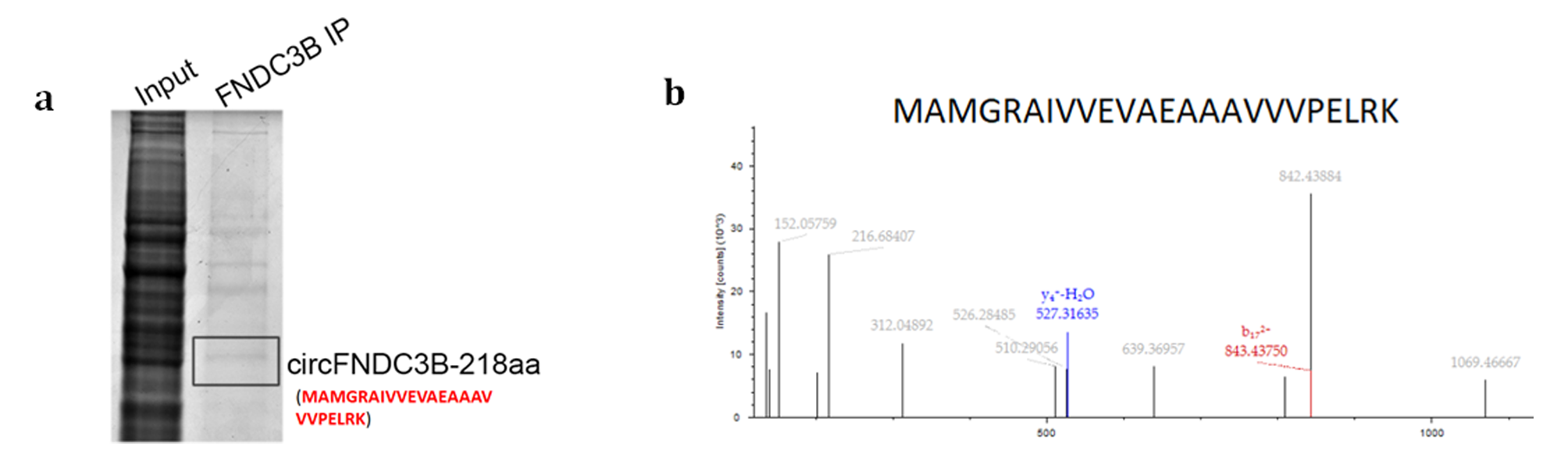

Supplement: Supplementary file 4 — Additional file 4: Figure S1. a The lysates from indicated cells were separated by SDS-PAGE. Protein at 25 kDa were excised manually and summited for identification by LC-MS/MS. b circFNDC3B-218aa specific peptide (MAMGRAIVVE VAEAAAVVVPELRK) was identified. [file 12943_2020_1179_MOESM4_ESM.tif]
